# Supplementary figures and images for: CAD Genes: Genome-Wide Identification, Evolution, and Their Contribution to Lignin Biosynthesis in Pear (Pyrus bretschneideri)
Source: Plants (Basel). 2021 Jul 15;10(7):1444. doi: 10.3390/plants10071444 (PMC8309377; doi:10.3390/plants10071444)

**The entire of sequence logos of motifs (Followed by motif 1 to motif 25)**

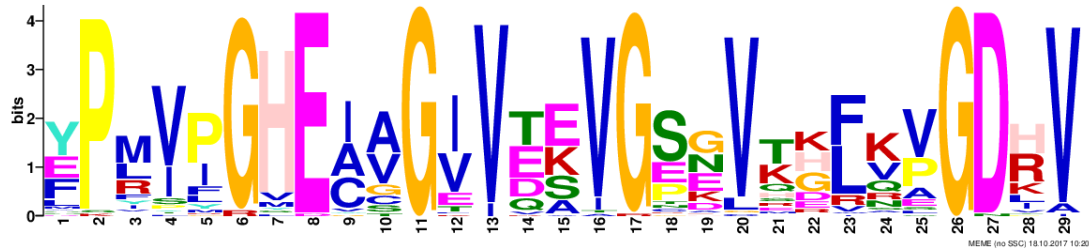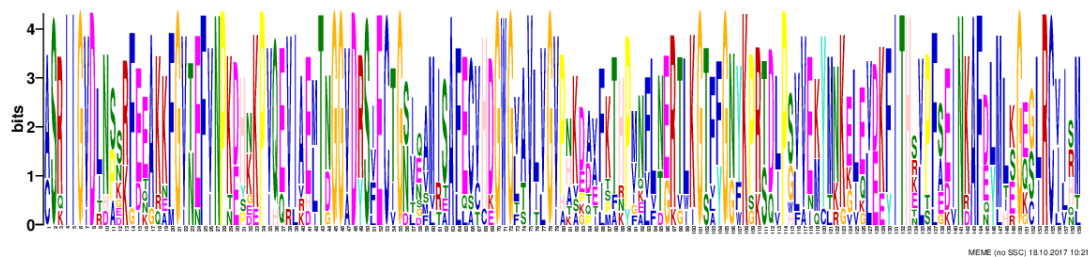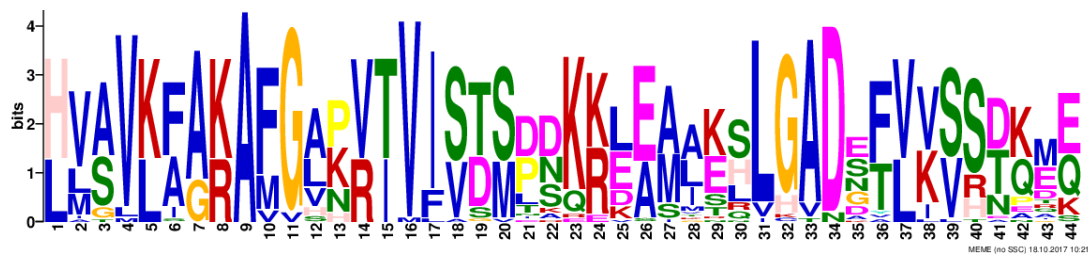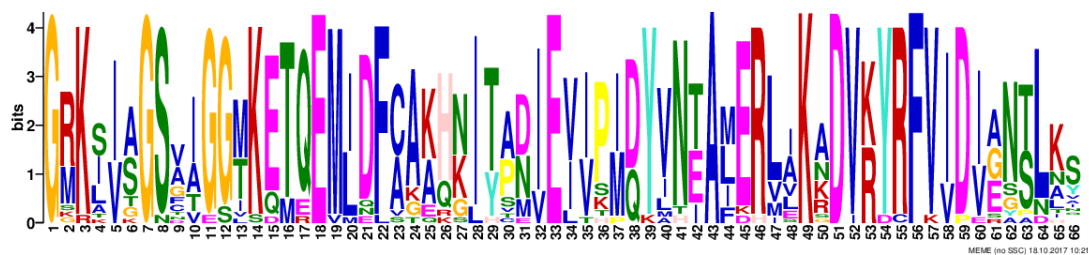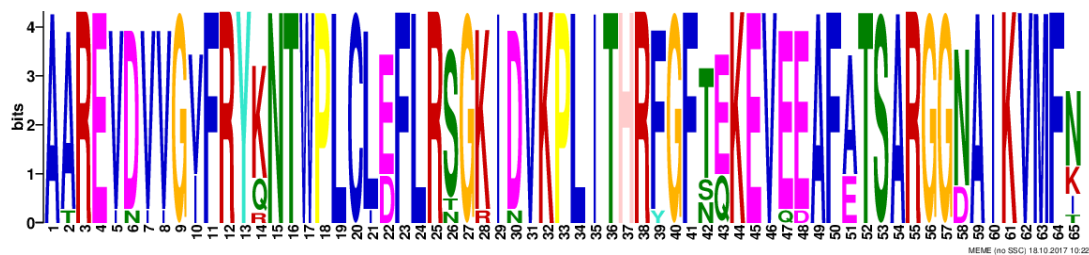

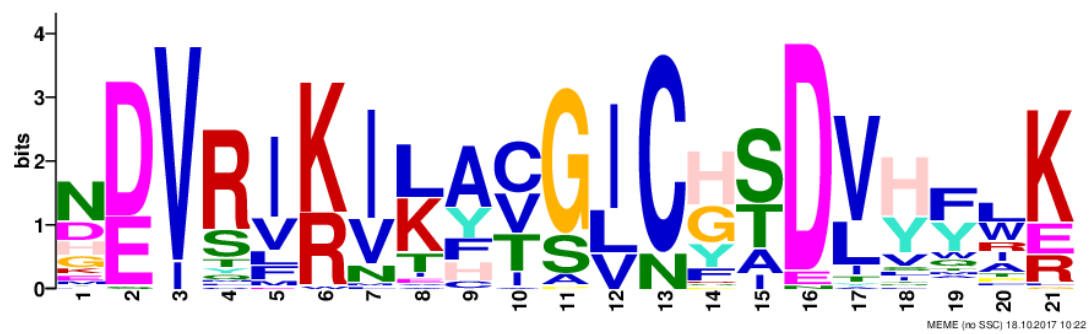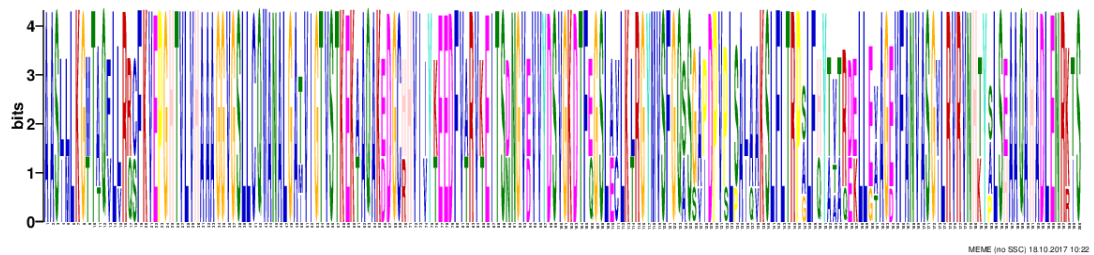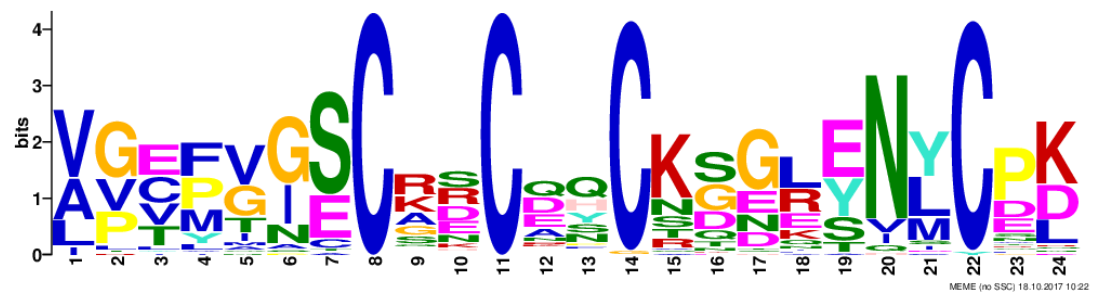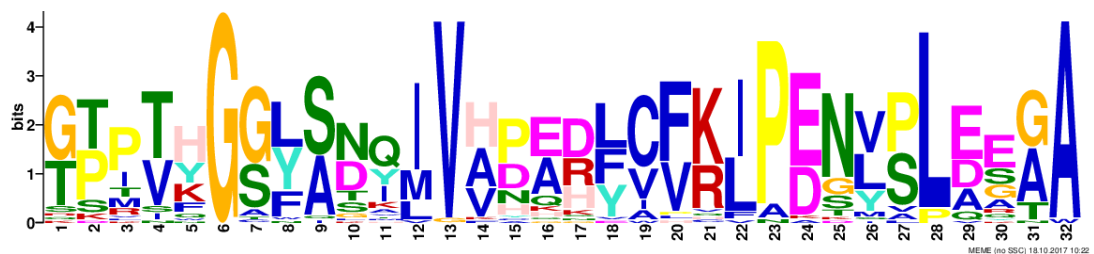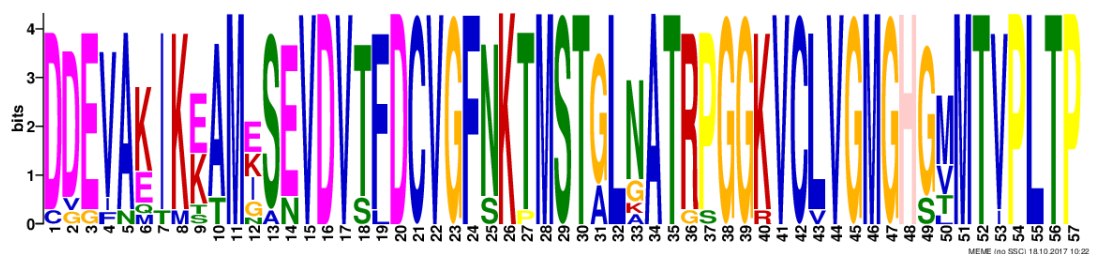

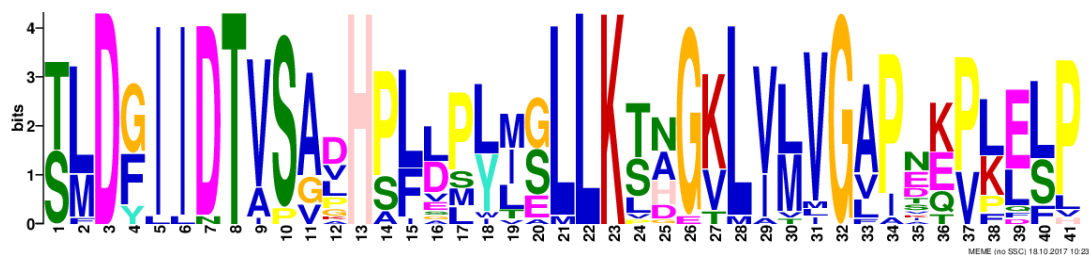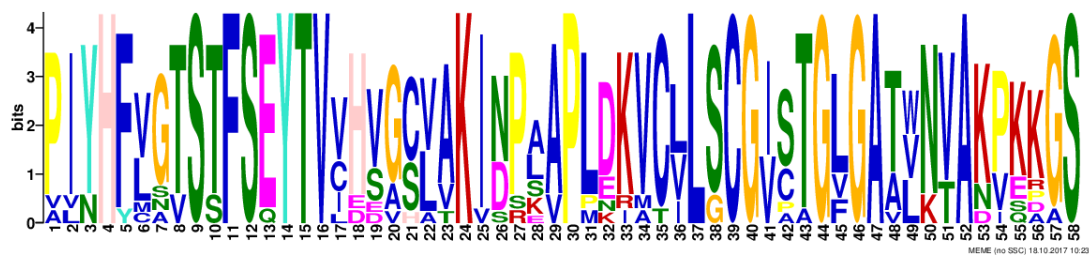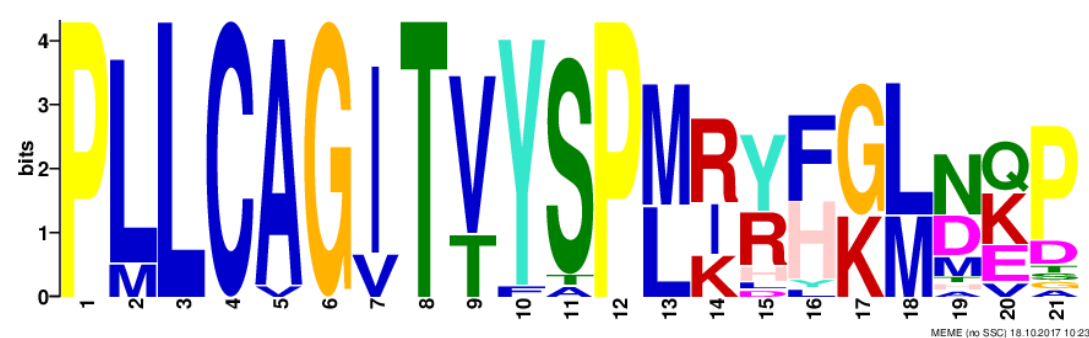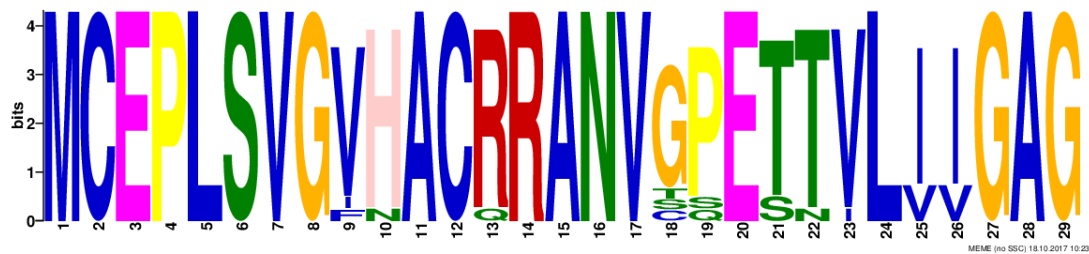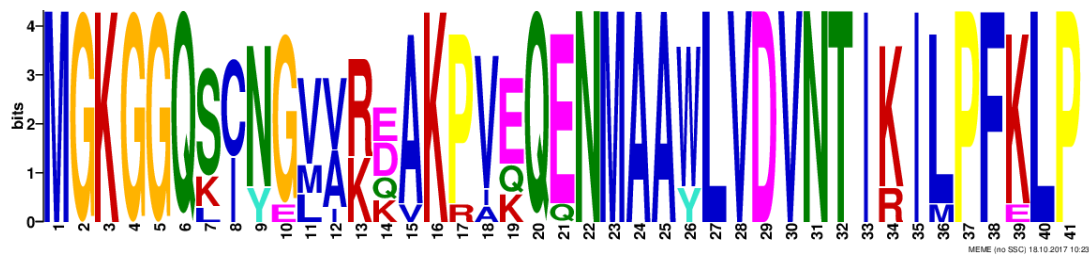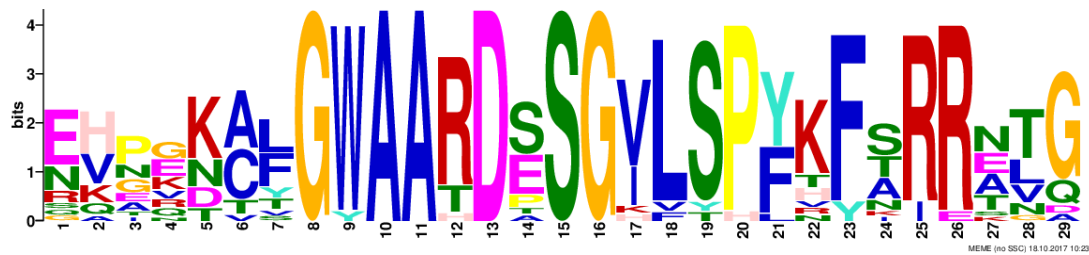

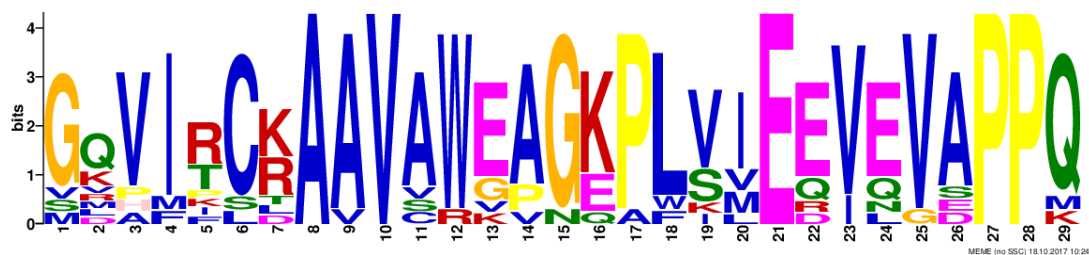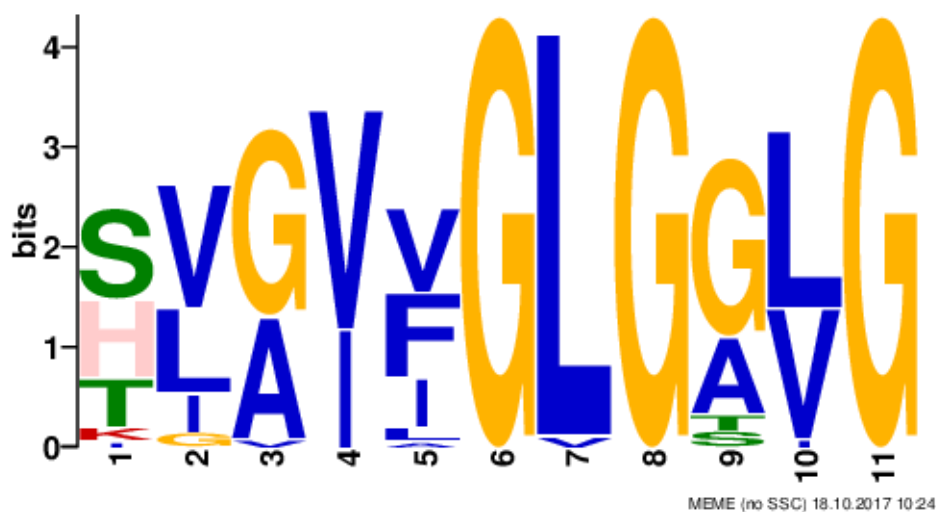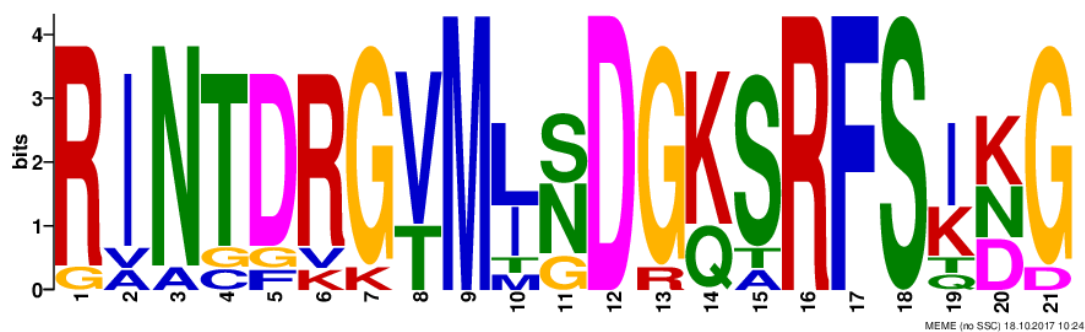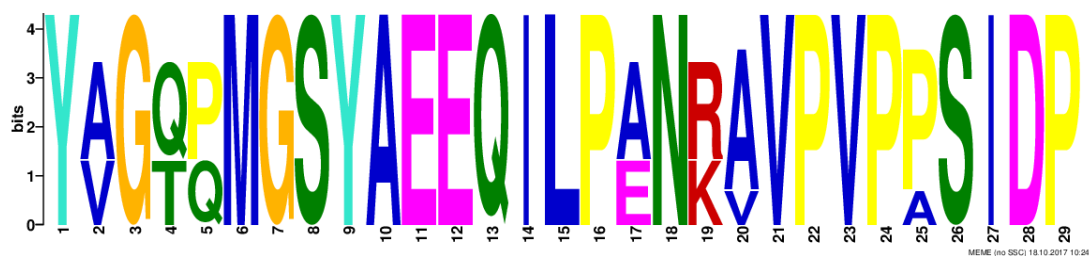

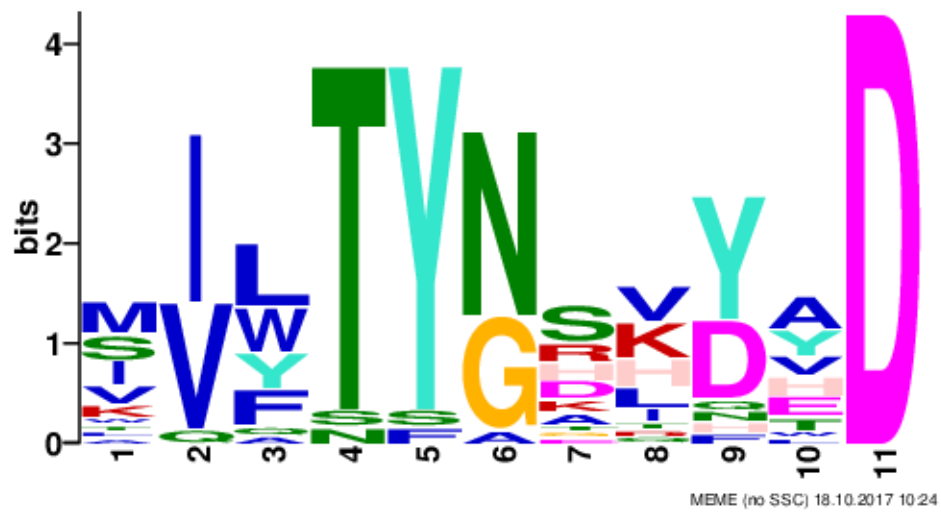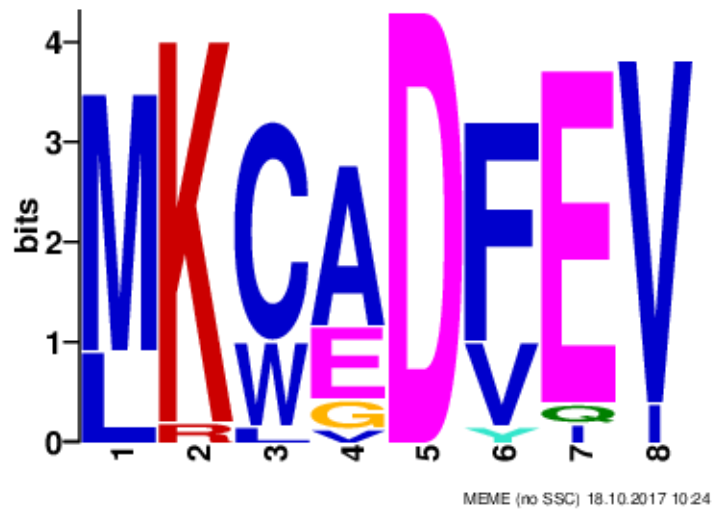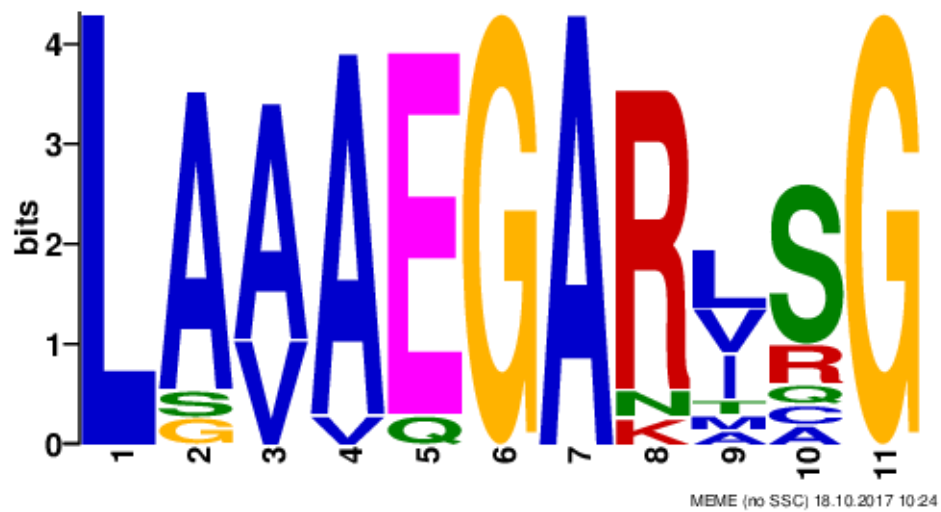

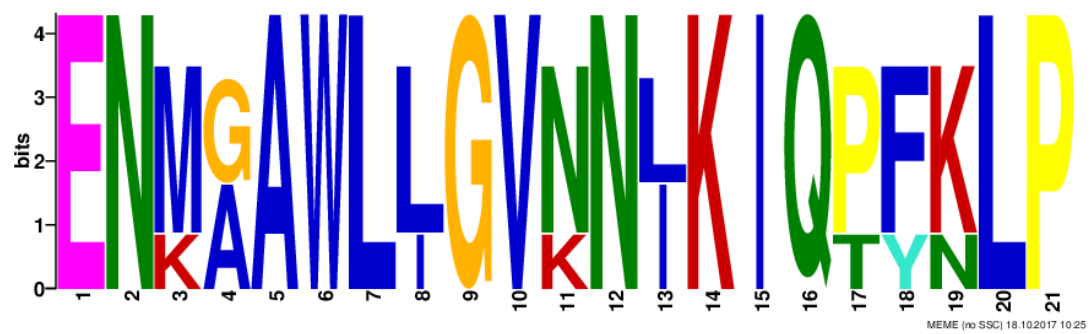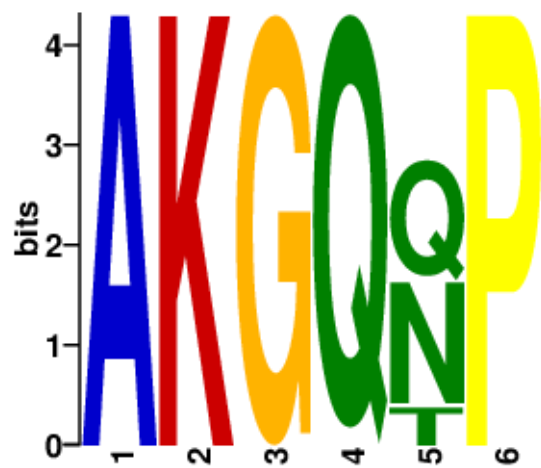

MEME (no SSC) 18.10.2017 10:25

Supplement: Supplementary file 1 [file plants-10-01444-s001.zip › Supplement tables/Table S5.pdf]
